# Application of conicity index adjusted total body fat in young adults-a novel method to assess metabolic diseases risk

**DOI:** 10.1038/s41598-018-28463-1

**Published:** 2018-07-04

**Authors:** Yujie Zhang, Qiang Zeng, Xiaoying Li, Pengli Zhu, Feng Huang

**Affiliations:** 1 Department of Geriatric Cardiology, Chinese PLA General Hospital, Beijing, China; 2 International Medical Center, Chinese PLA General Hospital, Beijing, China; 3 Department of Geriatric Medicine, Fujian Provincial Hospital, Fujian Institute of Clinical Geriatric, Fuzhou, China; 4 Fujian Medical University, Fuzhou, China

## Abstract

The aim of the study was to evaluate the usefulness of conicity index (CI) adjusted total body fat (TBF), which was defined as TBF/CI, in various metabolic diseases in young adults. A cross-sectional study was carried out in Chinese PLA General Hospital and a total of 1365 young adults (age 20–40 years) who underwent a health check-up examination were finally included in the analysis from February 2016 to 2017. Linear Regression and logistic regression were used to further examine relationship between the index and metabolic diseases. The average age was 34.5 years. *Odds Ratios* (*OR*s) for the risk of metabolic diseases increased from the lowest to highest TBF/CI quartile (all *P* trends < 0.001). Young adults with increased TBF/CI had higher risk of hyperhomocysteinemia (Hhcy) (*OR* = 1.528, *95% confidence interval* = 1.057–2.209). There was a 1.407 increase in the odds of obesity, a 1.112 increase in the odds of hyperlipidemia (HLP) and a 1.094 increase in the odds of diabetes mellitus (DM) per standard deviation (SD) increase in TBF/CI (all *P* < 0.001). TBF/CI showed higher predictive values for obesity, HLP, DM and Hhcy than weight adjusted total body fat (all *P* < 0.001). Young adults with increased TBF/CI had higher ratios of metabolic diseases, which suggested that TBF/CI can be a good indicator and had a close relationship with metabolic diseases.

## Introduction

As market aged with a booming economy and increasing material riches, metabolic diseases are becoming more prevalent among younger group. Scientists have paid more and more attention to the situation, but the evaluation markers were still outdated to assess the risk of metabolic diseases effectively.

Body Composition Analysis, namely bioelectric impedance (BIA), is largely based on the perspective that the electric resistance varies in different human body tissues, thus low-level and safe current which pass through tissues can be detected specifically, quickly and painlessly^1^. The technique has drawn more attention over the past years. Weight adjusted total body fat, measured by BIA, was closely associated with many diseases, including cardiovascular diseases, diabetes mellitus (DM) and osteoporosis *et al*.^2^.

Previous studies indicated that weight adjusted total body fat may be a better predictor of high blood pressure (HBP), hyperglycemia and hyperlipidemia (HLP), which exemplified cardiovascular risk factors, than body mass index (BMI)^1^. However, the value of it remains controversial. Several reports suggested that the trend of it and BMI differed in adults, thus there was no agreement about cut-off points for it to diagnose obesity^3^. Furthermore, the application of it is less convenient than BMI. Nevertheless, as the understanding of BIA gradually deepen, appendicular skeletal muscle mass index (ASMI), measured by BIA and then calculated by different formulas, have been used as indicators for the diagnosis of sarcopenia in the elderly people^4^. But the fomulas of weight adjusted body fat and ASMI could not consider the adipose distribution of participants well, even adjusted by BMI.

Conicity index (CI), which was a good tool to assess fat mass distribution and figure in young adults as previous studies suggested^5–7^. Even though CI adjusted indices have potential value in clinical application, little is known about the role of CI adjusted total body fat (TBF), namely TBF/CI, in metabolic diseases assessment. We firstly calculated the TBF/CI to determine whether it can be a good indicator and had a close relationship with metabolic diseases in young adults.

## Material and Methods

### Subjects

In total, 1365 ambulatory adult subjects aged between 20 and 40 years who underwent a routine health examination including BIA in Chinese PLA General Hospital from February 2016 to 2017 were enrolled. The inclusion criteria were young adults (age 20–40 years) who accepted BIA test. Participants were excluded for any of the following reasons: acute phase of chronic diseases, thyroid disorders, immune diseases, chronic renal failure, anorexia, malignant tumors, gastrointestinal surgery history and incomplete data. All participants were given oral informed consent to take part in the study and their data to be used. The study protocol was approved by the Institutional Review Board of Chinese PLA General Hospital. All methods were performed in accordance with the relevant guidelines and regulations.

### Data Collection and Measurements

Past history, personal history and family history were collected by trained healthcare providers. All subjects wore light clothes and stood in the upright position without shoes during BIA measurement using a body composition analyzer (X-Scan Plus-II, SELVAS Healthcare Inc., Geumcheon-gu, Seoul, Korea). Body mass, height, waist circumference (WC) and hip circumference (HC) were measured to an accuracy of 0.1(kg or cm) according to the standard protocol^8^. The 10 h fasting forearm vein blood was obtained in the early morning, and an Architect Ci8200-intergrated system (Abbott Laboratories, USA) was used to detect the level of blood glucose, whole-blood glycohemoglobin A1c (HbA1c), total cholesterol (TC), low-density lipoprotein cholesterol (LDL-C), high-density lipoprotein cholesterol (HDL-C) and total plasma homocystein (tHcy). Postprandial glucose was determined at 2 h after the administration of 75 g glucose.

### Diagnostic Definitions

BMI, waist-hip ratio (WHR), CI, percentage body fat (PBF) were calculated from the following equations:

$$\begin{array}{rcl}{\rm{BMI}} & = & {\rm{body}}\,{\mathrm{mass}/\mathrm{height}}^{{\rm{2}}};\\ {\rm{WHR}} & = & \mathrm{WC}/\mathrm{HC};\\ {\rm{CI}} & = & {\rm{WC}}/[0.109\,\times \,{\rm{square}}\,{\rm{root}}\,{\rm{of}}\,\mathrm{weight}/\mathrm{height}];\\ {\rm{PBF}} & = & \mathrm{TBF}/\mathrm{body}\,{\rm{mass}}\,\times \,{\rm{100}}{\rm{.}}\end{array}$$

In the present study, age, BMI, LDL-C, HbA1c and tHcy were incorporated into continuous metabolic risk variables. Smoking history was defined as daily smoking more than 1 and accumulating 100 or more cigarettes, cigars or pipes for at least 6 months^9^. Obesity was defined as BMI ≥ 28 kg/m^2^ and/or WC in male ≥ 90 cm while in female ≥ 85 cm^10^. HLP was defined as one of following: TC ≥ 5.2 mmol/L, LDL-C ≥ 3.4 mmol/L, HDL-C < 1.0 mmol/L, TG ≥ 1.70 mmol/L or previously diagnosed HLP^11^. DM was defined as fasting blood glucose ≥ 7.0 mmol/L, or blood glucose ≥ 11.1 mmol/L at 2 h after loading glucose, or HbA1C ≥ 6.5%, or previously diagnosed DM^12^. Hyperhomocysteinemia (Hhcy) was defined as tHcy ≥ 10 μmol/L or currently supplementing folate^13,14^.

### Statistical analysis

All the data were analyzed using the Statistical Package for the Social Sciences, version 22.0 (SPSS, Inc., Chicago, IL, USA). All continuous variables were approximately under normal distribution, and were expressed as mean (standard deviation (SD)). Categorical data were expressed as count (percentage). Linear trend tests were used to evaluate trends of TBF/CI changed in continuous risk variables (e.g. age, BMI, LDL-C, HbA1c and tHcy) and chi-quare trend tests were used in assessing *odds ratios* (*OR*s) of metabolic diseases varied with increasing quartile of TBF/CI. Moreover, linear regressions without adjustment were constructed to assess association of continuous risk variables with TBF/CI. Logistic Regressions were used to examine *OR*s of metabolic diseases identified by TBF/CI. Receiver Operator Curves (ROCs) were created to determine the predictive value of different anthropometric indices for various metabolic diseases. A two-sided *P* value < 0.05 was considered statistically significant.

## Results

### Participants’ Clinical Characteristics

The mean age was 34.5(5.0) years. TBF/CI in all the population, male and female were 14.99(5.25), 16.07(5.25), 13.22(4.76), respectively. 45.1%, 55.4%, 6.4% and 43.4% of young adults had obesity, HLP, diabetes and Hhcy, separately (Table 1).

**Table 1:** Participants’ clinical characteristics.

	Total (*n* = 1365)	male (*n* = 846)	female (*n* = 519)
Age (years)	34.5 (5.0)	34.8 (4.8)	33.9 (5.3)
Men (*n*, %)	846 (62.0)	—	—
Smoking history (*n*, %)	447 (32.7)	426 (50.4)	21 (4.0)
BMI (kg/m^2^)	24.56 (4.39)	26.32 (3.90)	21.69 (3.52)
WHR	0.84 (0.08)	0.89 (0.06)	0.76 (0.05)
HDL-C (mmol/L)	1.30 (0.39)	1.14 (0.30)	1.57 (0.39)
LDL-C (mmol/L)	3.03 (0.79)	3.18 (0.82)	2.80 (0.68)
TC (mmol/L)	4.66 (1.01)	4.78 (1.12)	4.46 (0.77)
HbA1c (%)	5.41 (0.72)	5.51 (0.85)	5.25 (0.38)
tHcy (μmol/L)	10.90 (6.29)	12.77 (6.75)	7.68 (3.58)
TBF/CI (kg^3/2^/m^3/2^)	14.99 (5.25)	16.07 (5.25)	13.22 (4.76)
Obesity (*n*, %)	615 (45.1)	513 (60.6)	102 (19.7)
HLP (*n*, %)	756 (55.4)	605 (71.5)	151 (29.1)
DM (n, %)	87 (6.4)	74 (8.7)	13 (2.5)
Hhcy (*n*, %)	592 (43.4)	500 (59.1)	92 (17.7)

Continuous data were shown as mean (SD) and categorical data were *n* (%).

BMI: body mass index; WHR: waist-hip ratio; HDL-C: high-density lipoprotein-cholesterol; LDL-C: low-density lipoprotein-cholesterol; TC: total cholesterol; HbA1c: hemoglobin A1c; tHcy: total homocystein; TBF/CI: total body fat/conicity index; HLP: hyperlipidemia; DM: diabetes mellitus; Hhcy: hyperhomocysteinemia.

### Trends of TBF/CI change in metabolic risk variables

**Figure 1:**
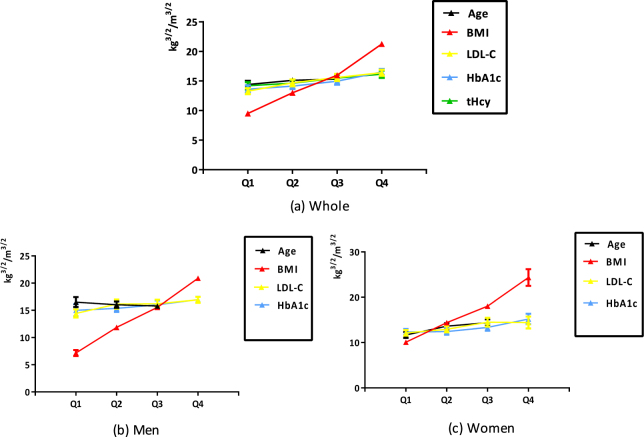
Trends of TBF/CI change in different metabolic risk variables. TBF/CI: total body fat/conicity index; BMI: body mass index; LDL-C: low-density lipoprotein-cholesterol; HbA1c: hemoglobin A1c; tHcy: total homocystein. Age: Quartile1: 20–33 years, Quartile2: 33–38 years, Quartile3: 38–40 years; BMI: Quartile1: <21.40 kg/m^2^, Quartile2: 21.40–24.20 kg/m^2^, Quartile3: 24.20–27.30 kg/m^2^, Quartile4: >27.30 kg/m^2^; LDL-C: Quartile1: <2.48 mmol/L, Quartile2: 2.48–2.98 mmol/L, Quartile3: 2.98–3.53 mmol/L, Quartile4: >3.53 mmol/L; HbA1c: Quartile1: <5.10%, Quartile2: 5.10–5.30%, Quartile3:: 5.30–5.50%, Quartile4: >5.50%; tHcy: Quartile1: <6.93 μmol/L, Quartile2: 6.93–9.25 μmol/L, Quartile3: 9.25–12.49 μmol/L, Quartile4: >12.49 μmol/L. Test for linear trend: *P* trend for age in whole = 0.010, other *P* trends < 0.001.

In whole, Increased TBF/CI was associated with increasing metabolic risk variables quartiles (all *P* trends < 0.001 except age quartile, *P* trend = 0.010). Besides, the increasing trends were also found in women and men respectively (all *P* trends < 0.001) except age quartile in men (*P* trend = 0.115) and tHcy quartile in both men and women (*P* trend = 0.565 in men and 0.207 in women) (Fig. 1).

### Association of metabolic risk variables with TBF/CI

**Figure 2:**
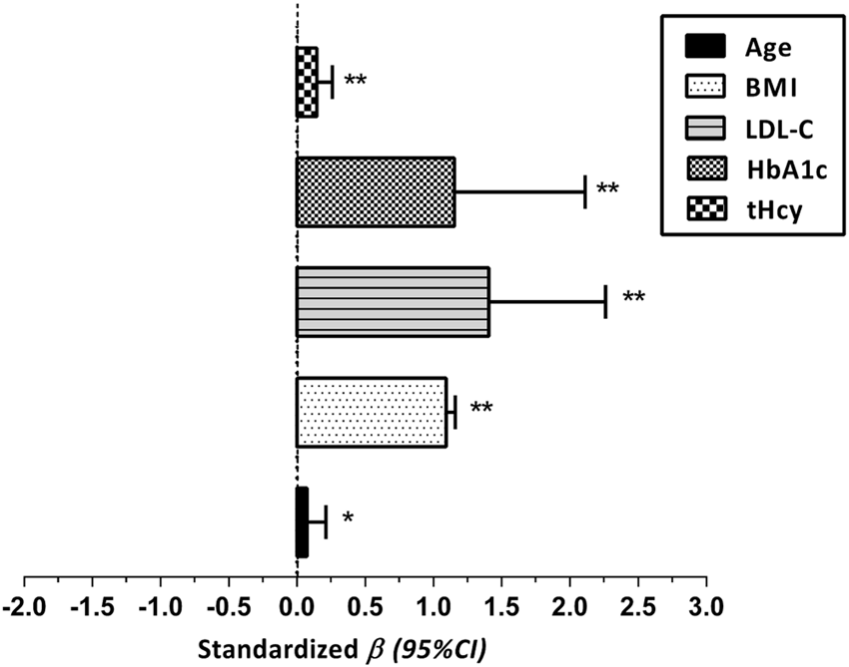
Association of metabolic risk variables with TBF/CI. TBF/CI: total body fat/conicity index; BMI: body mass index; LDL-C: low-density lipoprotein-cholesterol; HbA1c: hemoglobin A1c; tHcy: total homocystein. Linear Regression without adjustment: ^*^P < 0.05, ^**^P < 0.001.

Linear regression without adjustment demonstrated that age, BMI, LDL-C, HbA1c and tHcy were positively correlated with TBF/CI (*β* = 0.072 for age, 1.092 for BMI, 1.404 for LDL-C, 1.153 for HbA1c and 0.145 for tHcy; all *P* < 0.001 except age, *P* = 0.010) (Fig. 2).

### The odds of TBF/CI in metabolic diseases

In whole, multivariate-adjusted *OR*s for the risk of metabolic diseases increased from the lowest to highest TBF/CI quartile (all *P* trends < 0.001). Young adults with increased TBF/CI had higher risk of Hhcy (*OR* = 1.528, *95% confidence interval* = 1.057–2.209). There was a 1.407 increase in the odds of obesity, a 1.112 increase in the odds of HLP and a 1.094 increase in the odds of DM per SD increase in TBF/CI after adjusted for age, sex and smoking history (all *P* < 0.001).

Besides, the increasing *OR*s for the risk of Obesity, HLP, DM were also found in women and men respectively (all *P* trends < 0.001 except *P* trend for DM in men = 0.008 and 0.005 in women). Men with increased TBF/CI had higher risk of Hhcy (*OR* = 1.703, *95% confidence interval* = 1.072–2.706). There was a 1.528 increase in the odds of obesity in men, a 1.264 increase in women per SD increase in TBF/CI after adjustment (all *P* < 0.001). A 1.159 increase in the odds of HLP in men (*P* < 0.001) and a 1.055 increase in women (*P* = 0.008) were found as one TBF/CI unit increasing. The odds of DM increased 1.098 in men (*P* < 0.001) and 1.090 in women (*P* = 0.046) with TBF/CI changed per SD (Table 2).

**Table 2:** The odds of TBF/CI in metabolic diseases.

TBF/CI	Obesity	HLP	DM	Hhcy
Whole	1.407 (1.350–1.467)^††^	1.112 (1.084–1.142)^††^	1.094 (1.052–1.139)^††^	1.002 (0.977–1.027)
Q1	1.000 (reference)^**^	1.000 (reference)^**^	1.000 (reference)^**^	1.000 (reference)^**^
Q2	2.814 (1.810–4.375)^††^	1.257 (0.897–1.762)	1.546 (0.610–3.919)	1.185 (0.818–1.716)
Q3	8.658 (5.632–13.310)^††^	1.795 (1.274–2.528)^†^	2.789 (1.181–6.583)^†^	1.528 (1.057–2.209)^†^
Q4	43.965 (27.089–71.353)^††^	4.103 (2.846–5.914)^††^	4.092 (1.768–9.468)^†^	1.143 (0.791–1.652)
Men	1.528 (1.437–1.624)^††^	1.159 (1.118–1.201)^††^	1.098 (1.049–1.150)^††^	1.012 (0.983–1.041)
Q1	1.000 (reference)^**^	1.000 (reference)^**^	1.000 (reference)^*^	1.000 (reference)
Q2	4.020 (2.253–7.175)^††^	2.199 (1.392–3.475)^†^	2.012 (0.632–6.408)	1.204 (0.748–1.937)
Q3	15.973 (9.003–28.338)^††^	3.306 (2.101–5.204)^††^	3.559 (1.209–10.478)^†^	1.703 (1.072–2.706)^†^
Q4	88.950 (45.208–175.016)^††^	6.800 (4.218–10.962)^††^	4.459 (1.538–12.933)^†^	1.351 (0.866–2.107)
Women	1.264 (1.193–1.340)^††^	1.055 (1.014–1.098)^†^	1.090 (1.001–1.186)^†^	0.972 (0.921–1.025)
Q1	1.000 (reference)^**^	1.000 (reference)^**^	1.000 (reference)^*^	1.000 (reference)
Q2	1.770 (0.899–3.484)	0.697 (0.422–1.149)	0.789 (0.129–4.832)	1.232 (0.697–2.177)
Q3	2.806 (1.402–5.617)^†^	0.836 (0.482–1.450)	1.187 (0.193–7.311)	1.316 (0.704–2.459)
Q4	15.531 (7.718–31.254)^††^	2.584 (1.458–4.582)^†^	5.545 (1.317–23.351)^†^	0.582 (0.242–1.403)

Abbreviations: TBF/CI: total body fat/conicity index; HLP: hyperlipidemia; DM: diabetes mellitus; Hhcy: hyperhomocysteinemia.

TBF/CI: Quartile1: <11.44 kg^3/2^/m^3/2^, Quartile2: 11.44–14.51 kg^3/2^/m^3/2^, Quartile3: 14.51–17.87 kg^3/2^/m^3/2^, Quartile4: >17.87 kg^3/2^/m^3/2^.

Each of the independent variables in whole population has been adjusted for age, sex and smoking history. The variables in men and women have been adjusted for age and smoking history.

Test for linear trend: *P trend < 0.05, **P trend < 0.001; Logistic Regression: ^†^*P* < 0.05, ^††^*P* < 0.001.

### Predictive values of different anthropometric indices for various metabolic diseases

**Figure 3:**
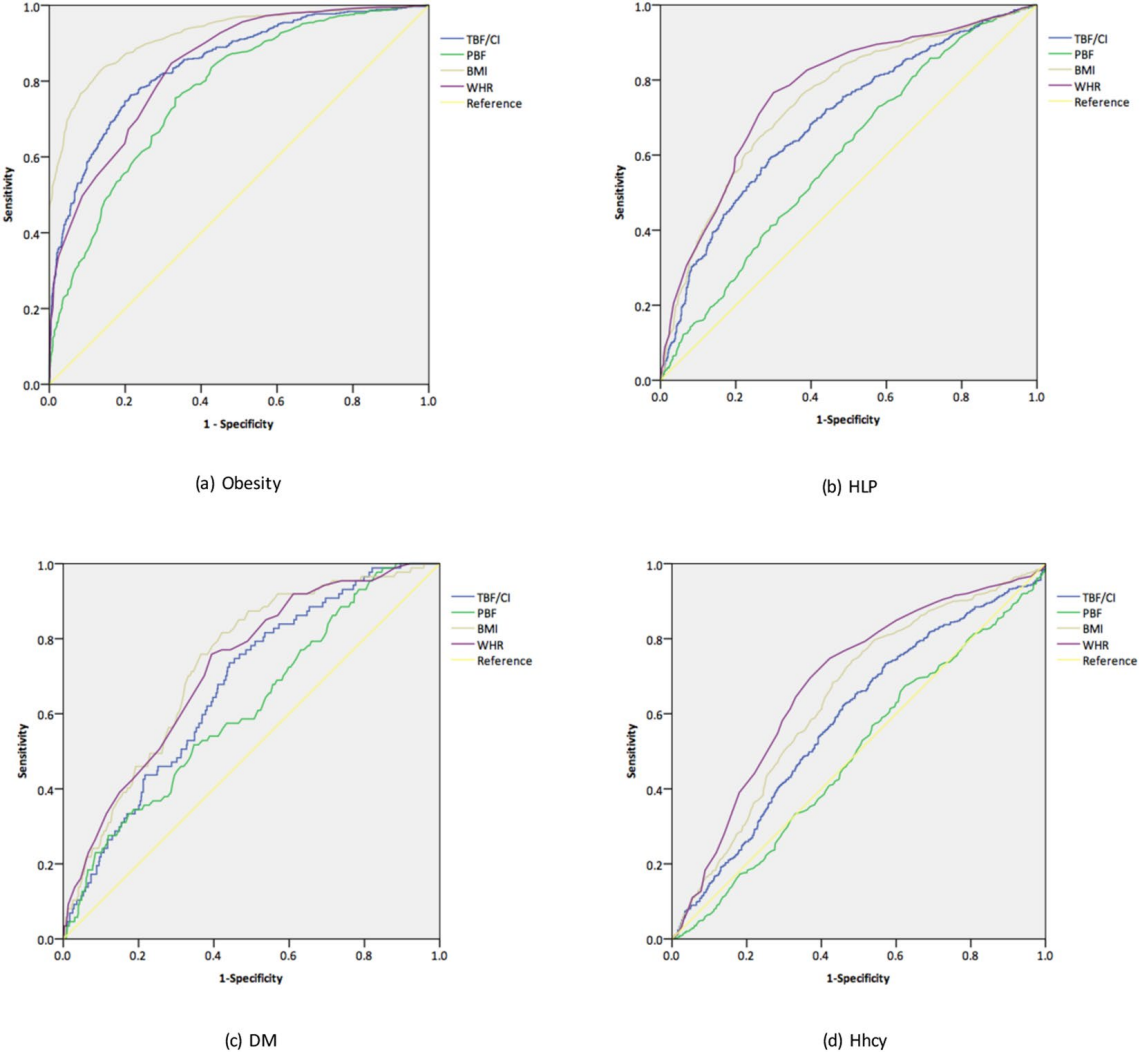
Receiver Operator Curves of different anthropometric indices as predictors of various metabolic diseases. TBF/CI: total body fat/conicity index; PBF: percentage body fat; BMI: body mass index; WHR: waist-hip ratio.

TBF/CI (*Area Under Curve (AUC)* = 0.845) showed a higher predictive value for obesity than WHR (*AUC* = 0.841) and PBF (*AUC* = 0.771), but lower than BMI (*AUC* = 0.921) (all *P* < 0.001). In HLP, DM and Hhcy, TBF/CI (*AUC* = 0.694 for HLP, 0.673 for DM and 0.587 for Hhcy) also displayed higher predictive values than PBF (*AUC* = 0.597 for HLP and 0.616 for DM, but *AUC* was not available to Hhcy because of *P* = 0.648), but lower than both BMI (*AUC* = 0.746 for HLP, 0.731 for DM and 0.643 for Hhcy) and WHR (*AUC* = 0.769 for HLP, 0.721 for DM and 0.682 for Hhcy) (all *P* < 0.001, Fig. 3). Cut-off value of TBF/CI for obesity was 14.91 kg^3/2^/m^3/2^ with 0.764 sensitivity and 0.784 specificity, for HLP was 13.86 kg^3/2^/m^3/2^ with 0.683 sensitivity and 0.601 specificity, for DM was 14.95 kg^3/2^/m^3/2^ with 0.736 sensitivity and 0.559 specificity and for Hhcy was 13.87 kg^3/2^/m^3/2^ with 0.653 sensitivity and 0.509 specificity.

## Discussion

Our study reported for the first time that the usefulness of TBF/CI in various metabolic diseases in young adults. CI is an useful and accuracy tool to evaluate the distribution of fat mass. As BMI cannot distinguish fat mass and lean mass clearly and its prediction is prone to be disturbed by the difference of samples in adipose distribution and body build, use of the BMI as a predictor for body fatness has been criticized^15^. However, TBF/CI, could represent the condition of total body fat well after balances individual adipose distribution. WHR, another anthropometric index, is not universally acknowledged as an accurate procedure to identify individuals at risk for metabolic syndrome^16^. As seen from in our study, TBF/CI showed a significant value. Linear Regression and logistic regression seperately demonstrated that TBF/CI had a higher correlation with tHcy and was in closer relationship with Hhcy. Moreover, as young adults with increased BMI did not display higher risk of Hhcy (*P* = 0.956, data not shown), TBF/CI can better evaluate the risk of Hhcy.

A cross-sectional study carried out in American middle age population^17^ demonstrated that intramuscular fat was associated with HLP risk. As mentioned above, we observed that the risk of HLP increased with added TBF/CI as well.

Even before-mentioned studies have pointed out that there were close ties between body composition and metabolic risk factors, controversy has emerged till now regarding the relevance between body fat and abnormal glucose metabolism. Canadian aged 20–69 and consisted of overweight or obesity were enrolled in a cross-sectional study by Kuk JL *et al*.^18^, which pinpointed that volatile fatty acid instead of whole-body skeletal muscle mass measured by magnetic resonance imaging was a significant predictor of abnormal glucose metabolism. Nonetheless, Son JW *et al*.^19^ did a prospective cohort study (follow-up 2 years) in South Korean community residents aged 40–69, which exhibited that a hazard ratio of 1.35 increase for the risk of developing type 2 diabetes per SD decrease in ASMI using multi-frequency BIA that strongly consistent with dual-emergency x-ray absorptiometry (DXA). In our present work, there were a 1.094 increase in the odds of DM per SD increase in TBF/CI. The results suggested that the diabetes risk increased with augmented TBF/CI in young adults and BIA maybe a viable, portable and accessible instrument for community and clinic-based assessment recommended by the Asian Working Group of Sarcopenia (AWGS)^20^. Disparate measurements in heterogeneous ethnic groups may account for the argumentative results^21^.

Accordingly, another association between body fat and tHcy has remained controversial. Battezzati A *et al*.^22^ elucidated that tHcy was positively related to fat-free mass and lean body mass in middle-aged Italian by a cross-sectional study. Whereas another study targeting Chinese hypertension population aged 45–75 years^23^ expatiated that nearly 20% decrease in OR for Hhcy in subjects with high physical activities. A significant positive correlation between tHcy and TBF/CI were displayed in our study. There maybe two possible explanations. For one thing, attention has been paid more to animal protein intake accompanied with fat intake which may lead to the ignorance of vegetable consumption in young’s diet^24^. As is known to us, vegetable was a vital and natural source of folate and vitamin B complex, and the lack of which could cause Hhcy. For another, the young was fit in a stage of good absorption and vigorous synthetic metabolism. Age, various health status, diet structure and surroundings may contribute to the disputable results^25^.

Generally speaking, TBF/CI has its irreplaceable advantages. Firstly, compared with biochemical test, TBF/CI was non-invasive. It was convenient and costless for early metabolic syndrome assessment^2,15^. Secondly, seen from our study, CI adjusted TBF showed higher predictive values for metabolic diseases than traditional definition, namely weight adjusted total body fat. Even the *AUC* of TBF/CI was less than the obesity gold standard-BMI, TBF/CI showed a higher predictive value for obesity than WHR. The function of TBF/CI is worth of further exploration in the future, maybe it could replace WHR sometimes especially in some patients who could not measure hip circumference for some special causes, like hip bedsore^26^. Thirdly, the *AUCs* of TBF/CI were nearly between 0.7 and 0.9, which manifested median value in assessing metabolic diseases.

However, as a cross-sectional study, the work had inevitable limitations. Firstly, biases were unavoidable since it was a single center research specific to check-up population. Secondly, we did not evaluate hormone level and nutritional status that could be confounding factors that affected TBF/CI. Last but not least, we only discussed the relationship between TBF/CI and metabolic diseases. So cause-and-effect relationship could not be demonstrated in the study. Therefore, a large and longitudinal cohort is warranted to further analyze the association between TBF/CI and metabolic diseases.

In conclusion, Young adults with increased TBF/CI had higher ratios of metabolic diseases, which suggested that TBF/CI can be a good indicator and exhibited a close relationship with metabolic diseases.
